# BAP1 in cancer: epigenetic stability and genome integrity

**DOI:** 10.1007/s12672-022-00579-x

**Published:** 2022-11-01

**Authors:** Sabrina Caporali, Alessio Butera, Ivano Amelio

**Affiliations:** Chair for Systems Toxicology, Department of Biology, University of Konstanz, 78464 Constance, Germany

## Abstract

Mutations in BAP1 have been identified in a hereditary cancer predisposition syndrome and in sporadic tumours. Individuals carrying familiar BAP1 monoallelic mutations display hypersusceptibility to exposure-associated cancers, such as asbestos-driven mesothelioma, thus BAP1 status has been postulated to participate in gene-environment interaction. Intriguingly, BAP1 functions display also a high degree of tissue dependency, associated to a peculiar cancer spectrum and cell types of specific functions. Mechanistically, BAP1 functions as an ubiquitin carboxy-terminal hydrolase (UCH) and controls regulatory ubiquitination of histones as well as degradative ubiquitination of a range of protein substrates. In this article we provide an overview of the most relevant findings on BAP1, underpinning its tissue specific tumour suppressor function. We also discuss the importance of its epigenetic role versus the control of protein stability in the regulation of genomic integrity.

## Gene, environment and BAP1

The largest majority, if not the entirety, of the human diseases emerge from the interaction of at least an exogenous factor, including also microorganisms, and the genetics of the individual. How the cell responds to a stress and manages to maintain the stability of its molecular circuits determines whether there will be a disease. Ability to regulate epigenetic landscape and the integrity of the genome is especially important in cancer development, hence, here, the gene-environment (GxE) interactions play critical roles [1–3].

Epigenetic modifications as DNA methylation and histone posttranslational modifications (PTMs) regulate cellular processes. Epigenetic perturbations or even mutations in epigenetic enzymes can trigger changes in chromatin conformation leading to aberrant transcriptome which may support tumorigenesis [4–6]. The balance between euchromatin and heterochromatin is finely tuned by a number of chromatin modifying factors, including the Polycomb group (PcG) family, broadly classified into 3 complexes: Polycomb Repressive Complex 1 (PRC1), Polycomb Repressive Complex 2 (PRC2) and Polycomb Repressive Deubiquitinase Complex (PR-DUB) [7]. BRCA1-Associated Protein 1 (BAP1) is a ubiquitin carboxy-terminal hydrolase (UCH), which also functions as a member of Polycomb Repressive—Deubiquitinase complex (PR-DUB). PR-DUB removes monoubiquitin residue at lysine 119 of the Histone 2A (H2AK119ub), thus remodelling chromatin and maintaining functional epigenetic landscape. This enzymatic activity directly counteracts Polycomb Repressive Complex 1 (PRC1)-mediated histone ubiquitylation, modulating transcriptional programs and a variety of cellular processes including DNA repair, metabolism, cell proliferation, differentiation and cell death [8–10]

**Fig. 1:**
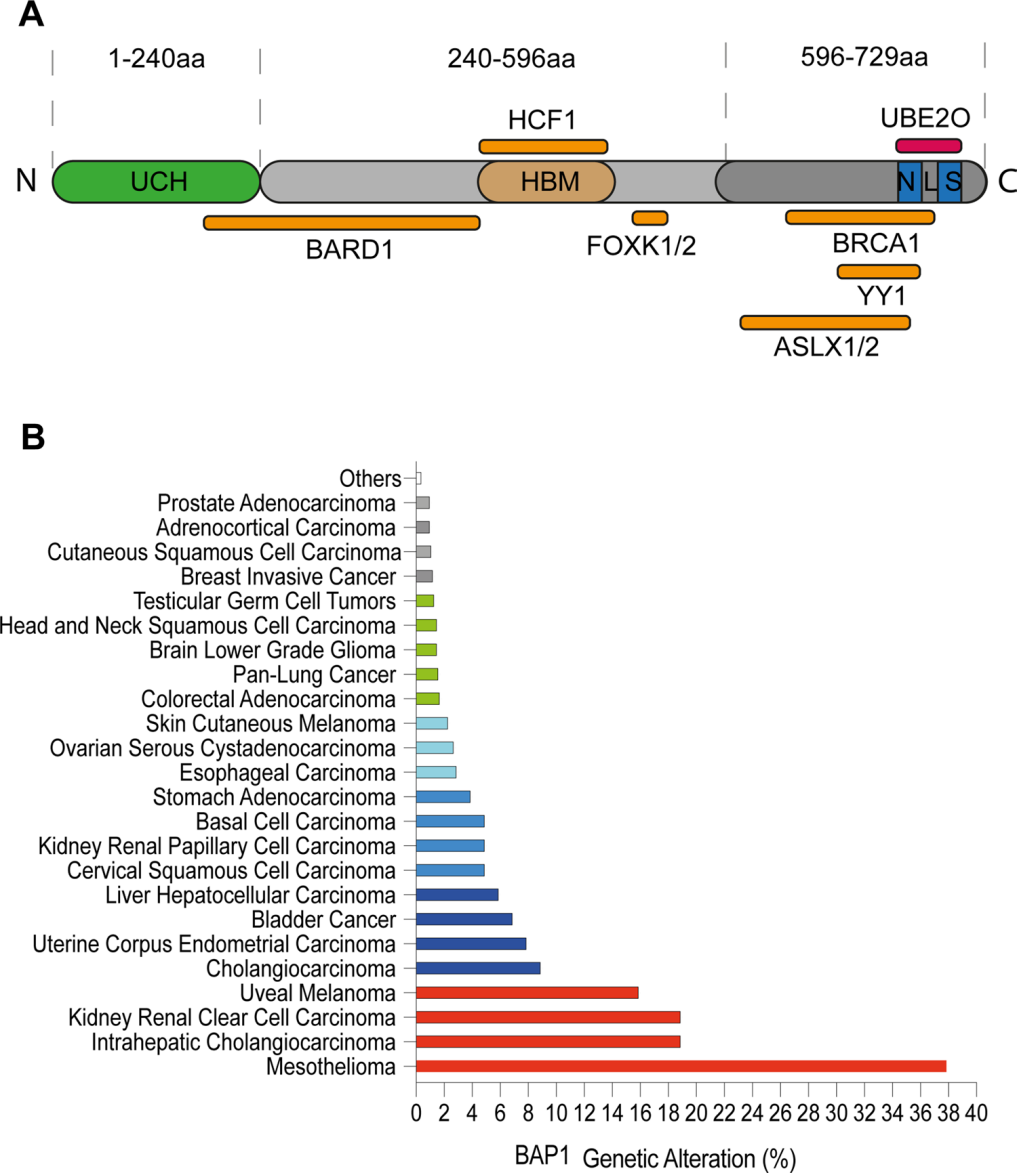
BAP1 structure and frequency of alteration in cancer. **a** Schematic representation of BAP1 structure and interacting partners. BAP1 protein can be divided in three regions: an N-terminal region (1–240 aa), where the catalytic triad is located and responsible for deubiquitylation; a middle region (241–596 aa), demonstrated to interact with BARD1, HCF1 through the HCF-1 binding motif (HBM) and FOXK1 and 2; the C-terminal region harbouring two nuclear localization sequences. The 2 NLS can be targeted by UBE2O allowing BAP1 retention in the cytoplasm. This region was demonstrated to interact with BRCA1, YY1 (Yin Yang 1) and ASLX1 and 2. **b** Frequency of genetic alteration of BAP1 in cancer. In red the most relevant alterations of BAP1 are shown. Mesothelioma, Intrahepatic Cholangiocarcinoma, Renal Clear Cell Carcinoma and Uveal Melanoma, display the highest frequency of BAP1 genetic alterations. Source: cBioportal [75]

Originally, BAP1 was directly implicated in a mechanism of DNA repair following double strand break. BAP1 direct physical interaction with BRCA1-RING finger domain was associated to enhancement of BRCA1 tumour suppressor activity in breast cancer [11] (Fig. 1a). BAP1-BRCA1 interaction is however still controversial [12]. Several studies later demonstrated that BAP1 mainly interacts with BARD1 perturbing BARD1/BRCA1 complex [13]. Nevertheless, further works have highlighted that BAP1 enables the initial recruitment and accumulation of BRCA1 and other DNA damage repair proteins as RPA and RAD51, at the double strand breaks (DSBs), requisite for DNA repair via Homologous Repair (HR) system [14–16].

BAP1 has attracted strong interest in the recent decades since the identification of the “BAP1 cancer syndrome”. Individuals carrying germline monoallelic mutation in BAP1 show a high frequency of malignant mesothelioma (MM), uveal melanoma (UM) and clear cell renal cell carcinoma (ccRCC) [15, 17, 18]. The germline mono-allelic mutation of BAP1 appears to play a role predisposition to exposure-induced cancers. This is particularly relevant in the context of asbestos-associated mesothelioma and UV-associated melanomas. Hence, such specific pattern of human cancers associated to BAP1 inactivation suggest a role in the response to environmental stressors and indicate a cooperation of predisposing gene mutations and environmental factors in cancer onset and progression. Thus, BAP1 mutation was proposed as key prototype of Gene-Environment interaction (GxE) [19]. Intriguingly, alike the canonical tumour suppressors such as p53 [20], sporadic mutations of BAP1 are found a peculiar spectrum of tumours, that recall the genetic predispositions (Fig. 1b). Hence, BAP1 inactivation emerged as directly linked to the tumorigenesis process of these cancers.

Despite the evidence that BAP1 enforces control of the epigenetic landscape and influences genomic integrity, it is still unclear whether the intersection of these two processes underlies a role of BAP1 in cancer. In this perspective, we discuss classic and more recent literature regarding BAP1, aiming to provide a unifying view of its role in epigenetic stability and genomic integrity.

## BAP1/PR-DUB: the fine regulation of histone ubiquitination

**Fig. 2:**
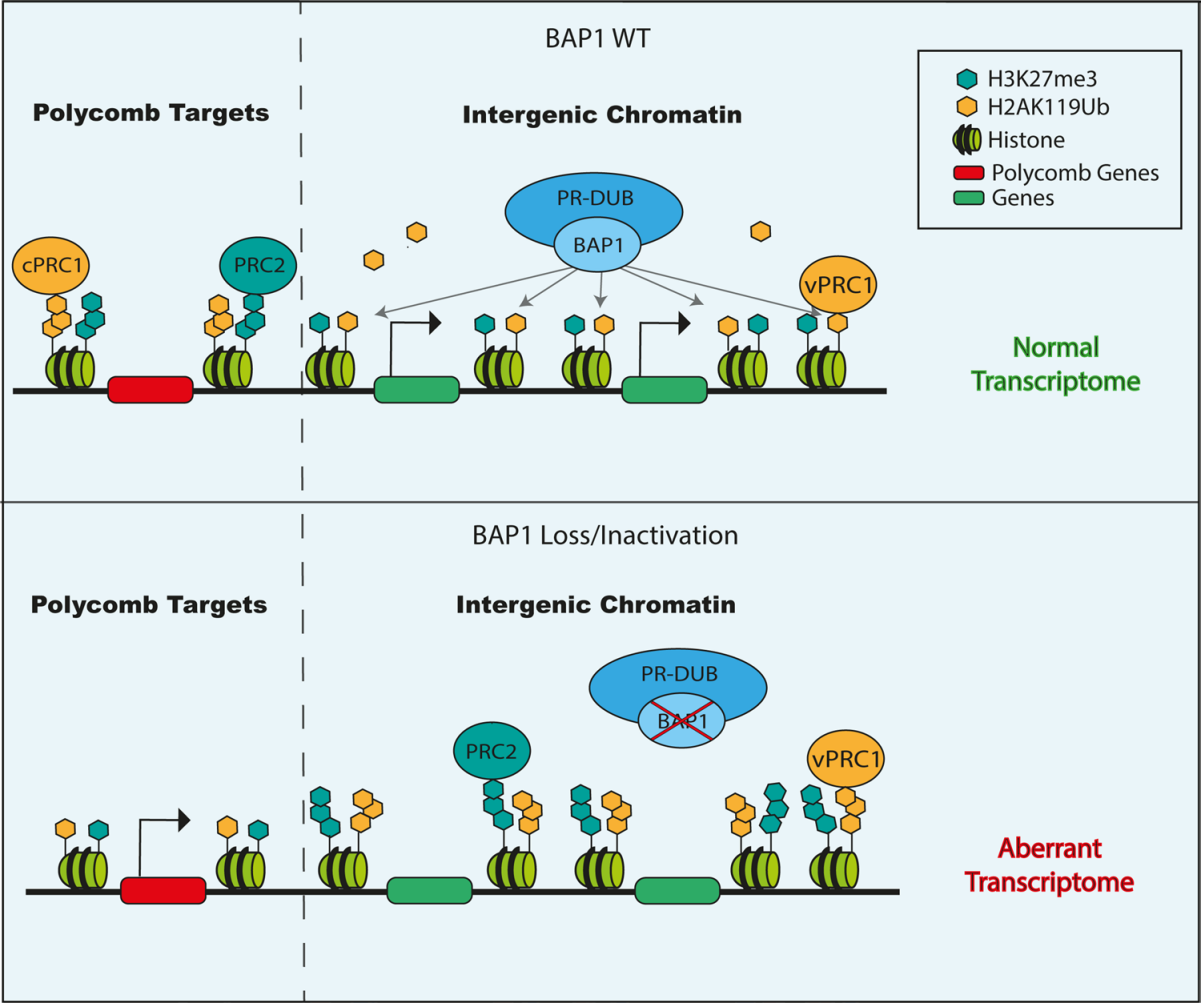
BAP1 in the PRC-DUB complex. BAP1 is directly involved in controlling the dynamics of chromatin as a component of PR-DUB complex. Loss of BAP1 directly impacts of cell transcriptome due to the altered deposition of H2AK119ub and H3K27me3 histone marks

The PR-DUB is a multiprotein complex constituted by BAP1, HCF1, FOXK1/2, OGT, MBD5/6, LSD2 associated with ASXL1, 2 or 3 [7, 21]. In this complex, BAP1 is the catalytic subunit and its activity is strongly dependent on the interaction with conserved DEUBiquitinase Adaptor (DEUBAD) domain of ASXLs that induces conformational changes able to increase BAP1 affinity for ubiquitin [22]. Moreover, FOXK1 and FOXK2 seem to have a role in site-specific recruitment of BAP1 across the genome [7] (Fig. 2).

The interplay between PRC1 and PRC2 complexes control the chromatin dynamic. Polycomb Repressive complex 1 monoubiquitylates H2A at lysine 119 (H2AK119ub), while Polycomb Repressive complex 2 catalyse mono-, di- and trimethylation of H3 at lysine K27 (H3K27me1-2-3), both posttranslational modifications of histone tails required for modulating chromatin architecture, cellular stemness and differentiation. An additional level of complexity is given by PR-DUB complex, which reshapes the epigenetic landscape by counteracting PRC1 activity through the removal of H2Aub from chromatin and, thus, indirectly influencing the PRC2-mediated H3K27me [7, 8, 23].

H2Aub is particularly enriched at specific silent genomic regions, notably Polycomb Target Genes (PcG) [22]. In mammalian, this group includes 39 Hox genes located in 4 clusters, involved in vertebrate development and organogenesis and their deregulation is commonly observed in cancer [24, 25]. Generally, Hox genes are silenced via two different mechanisms: the methylation of CpG islands in their promoters and PRC1/PRC2-driven chromatin repression by ubiquitination of H2A and trimethylation of histone H3 at lysine 27. RNF1/RNF2-mediated ubiquitylation of histone H2A is a required step for the recruitment of PRC2 on chromatin thereby leading to the subsequent deposition of H3K27me3 [22]. BAP1/PR-DUB associates to chromatin at active gene promoters and removes ubiquitylation mainly deposited by PRC1.3/5 (non-canonical PRC1 complex) while it is excluded from the canonical PRC1 (c-PRC1) and PRC2 Polycomb repressive regions [8]. Overall, BAP1 maintains the spatial distribution of both H2AK119ub and H3K27me3 on Polycomb regions, preserving gene repression. Indeed, loss of BAP1 catalytic function titrates away both c-PRC1 and PRC2 complexes from their genomic loci causing derepression of Polycomb genes, the spreading and accumulation of H2AK119ub and H3K27me3 sustained by PRC1.3/5 at intergenic sites across the genome [8]. This results in a global chromatin compaction, reshaping of epigenetic landscape and aberrant transcriptome that associates with loss of cellular identity, oncogenesis, immune evasion and poor tumour response to immunotherapy [26–28] (Fig. 2). Indeed, Yan and colleagues have recently demonstrated that the unbalanced activity of chromatin modifying factors like that caused by PRC2 inactivation, can strongly contributes to epigenetic reprogramming and transcriptional downregulation of genes involved in the immune cell recruitment, driving immune-desert tumour microenvironment [28]. Thus, identification and targeting of tumour-specific epigenetic dysregulations represent a possible therapeutic approach via administration of epidrugs as EZH2 (inhibitors of histone methyltransferase, core component of PRC2 complex), DNMT (DNA Methyltransferase), HDAC (Histone deacetylase) and BAP1 inhibitors [18, 29–31].

## BAP1 in cancer: more than epigenetic?

Several studies have highlighted that BAP1 tumour suppressor activity is strictly cell type- and context-dependent: inactivation of BAP1 or catalytically inactive mutants (i.e. C91A) can drive opposite phenotypes in different tissues [12, 32, 33].

BAP1 conditional knock out mice showed hematopoietic defects as anaemia, thrombocytopenia, leucocytosis, liver damage and atrophy of pancreas. In these organs, increases in cleaved caspase-3 levels suggest that the loss of this protein triggers apoptotic events. Furthermore, BAP1 loss-associated lethality can be observed in several cell types including embryonic stem cells, primary keratinocytes and E1A-immortalized embryo fibroblasts [31]. Conversely, BAP1 genetic deletion in mouse primary melanocytes and mesothelial cells induces proliferation and the expression of pro-survival genes. The differential activity in cell types resides into the different selectivity of BAP1 in regulating gene expression [32, 34]. The tumour suppressor function of BAP1 is also described in prostate and kidney cancers in which BAP1-dependent deubiquitinase activity stabilizes the tumour suppressor Phosphatase and Tensin homolog (PTEN) and Death Inducer-Obliterator 1 (DIDO1), a protein of the centrosome involved in spindle assembly and correct chromosome segregation [35–37]. In pancreas, BAP1 inactivation causes organ atrophy while triggers the inactivation of tumour suppressor Hippo pathways in pancreatic KRAS mutated cancer [38, 39]. Hence the interplay between BAP1 deficiency and oncogenic KRAS leads to pancreatic tumour progression. In breast cancer cell lines, BAP1 plays an oncogenic function by directly deubiquitinating and stabilizing KLF5 (Kruppel-like factor 5). Protein stabilization of KLF5 promotes cell proliferation, migration and tumour growth [38]. Moreover, in these KLF5-positive cell lines, BAP1 knock-down inhibits the DNA synthesis reducing cell viability, while it has no effect on the cell growth in KLF-5 negative MDA-MB-231 breast cancer cells [21, 41]. In small cell lung carcinoma (SCLC), BAP1 promotes oncogenic roles inducing the expression of ASCL1 (Achaete-Scute Family BHLH Transcription Factor 1), a key lineage-specific oncogenic driver in SCLC. BAP1 inhibitors and CRISPR-cas9 knock-out in NCI-H1963, NCI-H748 and NCI-H1882 cells abrogate ASCL1 chromatin occupancy at the promoter region of its target genes reducing cell growth [18]. In leukaemia the gain-of-function of ASXL1 mutants increase PR-DUB activity; the stabilization of BAP1 and its undue ASXL1 mutant-dependent chromatin recruitment leads to aberrant oncogenic pattern of gene expression [42, 43]. In this context, the reduction of BAP1 catalytic activity with iBAP (BAP1 inhibitors) might represent a therapeutic strategy [40]. In contrast, BAP1 function is required to avoid the onset of myeloproliferative disorder since BAP1 KO mice showed hematopoietic defects as myeloid progenitor expansion [44, 45].

BAP1 exerts its function predominantly in the nuclear compartment as the two NLS can direct the translocation of this protein in the nucleus. Notably, BAP1 can be sequestered in the cytoplasm by the ubiquitin-conjugating enzyme UBE2O that ubiquitylates its nuclear localization signals leading to its cytoplasmatic retention. Exogenous and endogenous stimuli trigger the activation of the self-deubiquitylation activity of BAP1. This allows the translocation from cytoplasm into nucleus where BAP1 regulates the cell biological response [44]. Furthermore, BAP1 exerts cytoplasmatic functions as it can localize in the endoplasmic reticulum for modulating intracellular Ca ^2+^ release and the activation of apoptosis [45]. BAP1 regulates stabilization of type-3 inositol-1,4,5-trisphosphate-receptor (IP3R3), ER calcium channel that controls the release of Ca2 + from endoplasmic reticulum into cytosol and mitochondria. Changes in mitochondrial permeability are required for the activation of the apoptotic process and the loss of BAP1 protects the cells from caspase-induced cell death as a consequence of IP3R3 level reduction and Ca2 + signalling decrease [45]. In addition to apoptosis, BAP1 also modulates the activation of cysteine-dependent cell death, ferroptosis, by downregulating the expression of SLC7A11, the major transporter for extracellular cysteine uptake. Also in this context, the inactivation of BAP1 triggers cell resistance to ferroptosis [10]. Moreover, BAP1 deficiency drives the reprogramming of cell metabolism, promoting anaerobic glycolysis for energy production rather than mitochondrial respiration and increasing extracellular lactate secretion which induces immune evasion, tumour growth and cell malignant transformation.

BAP1 emerges as a highly tissue-specific and context-specific tumour suppressor participating to the biology of the tumour with multiple mechanisms and different levels (summary in Table 1).

**Table 1:** Cell type and tissue-dependent functions of BAP1

Model	Tissue	Effects of BAP1 Loss/Inactivation	References
*Rosa26 CreER*^*T2*^* Bap1*^*iC19A/ fl*^* mice*	Embryonic Stem Cell (ES)	BAX/BAK dependent- Apoptosis	[31]
	Embryo Fibroblasts (MEFs)	Apoptosis	
	Primary Keratinocytes		
	Primary Melanocytes	Proliferation/Melanocyte differentiation. Up-regulation of melanoma oncogene *Mitf*	
	Primary Mesothelial cells	Proliferation	
*Bap1 *^*fl/fl*^* Kras lsl/* + *Pdx1.cre mice*	Cell line derived from KRASG12D/BAP1KO tumours	Deregulation of tumour suppressor Hippo pathway by LATS2 decrease which causes upregulation of downstream YAP1 and TAZ oncoproteins	[36]
*Rosa26 CreER*^*T2*^* Bap1*^*fl/ fl*^* mice*	BAP1-KO Hematopoietic progenitor cells	Proliferation and cell cycle progression in myeloid progenitors	[43]
*Asxl1*^*Y588X*^* Transgenic mice*	Hematopoietic Asxl1^Y588x^ progenitor cells	Reduction of BAP1 activity prevents ASXL1 mutants -driven myeloid malignancy	[41]
*Mx1-Cre-BAP1*^*fl/fl*^* mice*	BAP1-deficient Hematopoietic progenitor cells	Proliferation and cell cycle progression in myeloid progenitors	[42]
*BAP1*^*fl/fl*^* Nf2 *^*fl/fl*^* Cdkn2ab *^*−/−*^*mice*	Malignant mesothelial cells	EZH2/PRC2-mediated H3K27me3 global redistribution at gene promoter sites Activation of P13K/Akt and MAPK/ERK signalling pathways	[33]
		BAP1 deletion increases sensitivity to γ-radiation and PARP inhibitor AZD2461	
*Pax8-Cre Vhl *^*fl/fl*^* BAP1*^*fl/*+^ *mice*		Mutations in BAP1 and VHL cooperate in tumour development. Loss of BAP1 is associated to high grade of tumour and hyperactivation of mTORC1	[68]
*Human Breast Cancer*	HCC1806 HCC1937 MDA-MB-468	KLF5 ubiquitination. Growth arrest/ Cell Viability decrease	[39]
*Human Clear Cell Renal Cell Carcinoma*	293 T 786-O HK-2	Downregulation of MCRS1 protein, required for spindle assembly and chromosome segregation causing genome instability and aneuploidy	[67]
		Downregulation of DIDO1 protein leading to aberrant mitotic spindle and chromosome instability	[35]
	Patient-derived BAP1 mutant renal tumour cells	Lack of BAP1 sensibilizes to Anti-CCR5 (Maraviroc) treatment leading to necrosis of renal tumour cells	[69, 69]
*Human Head and Neck Cancer*	HN31 (HP negative, p53 mut) UMSCC47 (HP-positive, p53 WT)	Increases cell sensitivity to irradiation caused by the impairment of BAP1-dependent DNA damage repair systems	[16]
*Human Intrahepatic Cholangiocarcinoma*	HCCC-9810 (Low BAP1) RBE (high BAP1)	Loss of BAP1 enhances cell proliferation, and invasion	[72]
*Human Leukaemia*	ASXL1-Y591fs-THP1 cells	Reduction of BAP1 activity prevents ASXL1 mutants -driven leukaemia	[40]
*Human Melanoma*	Mel202, 92.1	DNA methylomic repatterning, deregulation of genes related to axon guidance and melanogenesis pathways	[73]
*Human Mesothelioma*	*Patient-derived BAP1* ± *fibroblasts*	HDAC1 ubiquitination. Extracellular secretion of hyperacetylated HMGB1 triggering inflammation process and cell transformation	[74]
*Human Prostate Cancer*	DU145	PTEN ubiquitination. Malignant cell growth by Akt signalling pathway	[34]
*Human Small Cell Lung Carcinoma*	NCI-H1963 NCI-H748 NCH-1882	Reduction of ASCL1 activity, key lineage-specific oncogenic driver	[18]
*Human Uterus/Cervix Adenocarcinoma*	HeLa	PTEN ubiquitination. Malignant cell growth by Akt signalling pathway	[34]

## The BAP1 cancer syndrome

Germline monoallelic inactivation of BAP1 is a prototype of GxE predisposing to tumorigenesis. Carriers of BAP1 mutations have high frequency of mesothelioma, cutaneous and uveal melanoma, clear cell renal cell carcinoma. In carrier individuals, tumour onset is accompanied by the loss of heterozygosity with the inactivation of the second wild-type allele [12]. More than 80% of gene carriers are affected by at least one type of cancer and 90% of the affected individuals have at least two close first-degree relatives affected by a cancer. BAP1 families require genetic and oncological counselling to handle cancer risk management and undergo routine testing for at-risk family members.

Tumour onset in carriers is accompanied by the loss of heterozygosity with the inactivation of the second wild-type allele [12]. Mutations frequently occur in the N-terminal catalytic UCH domain within Gly185, Arg227, impacting the affinity of BAP1 for ubiquitin, and within Cys91, His169, Asp184 by inactivating the catalytic domain. Missense mutations are also found in BAP1 interacting domains and in C-terminal region, interfering with its nuclear localization, auto-deubiquitination and recruitment on chromatin [12, 46, 47].

Therapeutic approaches have been suggested for the treatment of BAP1-deficient cancers such as the epigenetic drugs that inhibit EZH2, ther platinum-based compounds and PARP-1 inhibitors. EZH2 inhibitors reduces proliferation of BAP1-mutant mesothelioma cell lines, while platinum-based drugs and PARP-1 inhibitors should be able to target cancer cells with defective DNA repair mechanisms [9, 44, 48].

Here, we discuss the current knowledge about the contribution of inactivating BAP1 mutations in development and progression of inherited cancers as Mesothelioma, Uveal melanoma and Clear Cell Renal Cell Carcinoma in which BAP1 is recurrently lost.

### Mesothelioma

Malignant Mesothelioma (MM) is a tumour arising from mesothelial cell transformation mainly of pleura and peritoneum and it is correlated to persistent exposure to environmental carcinogen such as asbestos that includes 6 natural fibres (crocidolite, actinolite, tremolite, anthophyllite, amosite and chrysotile) [17]. After inhalation, asbestos is phagocytized by macrophages and mesothelial cells of pleura where accumulates forming deposits and hence exerts its cytotoxic effects [47]. The initiation of carcinogenesis process is attributed to HMGB1 extracellular release by necrotic mesothelial cells that activates chronic inflammation and ROS production triggering an inflammatory microenvironment [48]. In addition, these fibres could mechanically interfere with chromosomal segregation during mitosis leading to DNA damage, genome instability, thus contributing to mesothelial cell oncogenic transformation [49, 50]. In addition to mutations of BAP1, frequent deletion of tumour suppressors Cdkn2a/b and Nf2 were observed in malignant mesothelioma [51–53]. The functional interaction between BAP1 inactivation and these other genetic events in the development of MM has not been fully elucidated.

### Uveal melanoma

Uveal Melanoma (UM) is the most widespread primary intraocular malignant tumour in adult arising from melanocytes of pigmented uveal tissues as the iris in the anterior chamber of the eye and ciliary body and choroid in the posterior chamber of the eye [54, 55]. UM incidence shows a south to north increasing gradients as it ranges from < 1 (Africa) up to 9 (Norway and Denmark) per million population per years depending on the countries [54]. Despite the treatment with radiotherapy until the ocular enucleation in most advanced cases, the half of patients affected by UM develop metastases within 5-years in liver, lung, skin and brain reducing the survival at less of one year from the onset of symptoms [55]. Genetic features as fair-skin, light-coloured eyes, ocular melanocytosis besides germline mutations in BAP1 gene, increase the odds of developing uveal melanoma [12, 56, 57]. Moreover, loss of chromosome 3 or BAP1 deficiency are predictors of metastatic UM since BAP1 biallelic inactivation correlates with the most aggressive phenotype of melanocytes, characterized by driver mutations in G-protein-α subunits GNAQ or QNA11 [25, 30, 58, 59] that are not sufficient alone to induce malignant transformation but sustain cell growth by downstream activation of YAP/TAZ [60–62]. However, the molecular mechanisms through which BAP1 promotes UM metastasis is still unclear.

An important open question is whether the sunlight exposure could cooperate with BAP1 inactivation in UM development and progression. Interestingly, few evidences indicate a direct link between ultraviolet radiation exposure, a common environmental risk factor for cutaneous melanoma onset, and the occurrence or progression of uveal melanoma [56, 63]. Nevertheless, the melanoma of the iris, the part of the eye directly exposed to sunlight, shares ultraviolet radiation (UVR) mutational signature, suggesting an association between UV exposure and this malignancy [64, 65].

### Clear cell renal cell carcinoma

Clear cell renal cell carcinoma (ccRCC) represents 70–80% of all kidney tumours and arises from epithelial cells of renal tubular. ccRCC is characterized by genetic features as loss of chromosome 3p, mutations in Protein Polybromo-1 gene (PBRM1), Von Hippel–Lindau (VHL), Set domain-containing 2 (SETD2) histone methyltransferase and BAP1 genes [66, 67]. BAP1 inactivation status is strongly associated to high tumour grade and worse clinical outcomes in ccRCC patients [68–70]. However, BAP1 mutations increase the susceptibility of renal cancer cells to the treatment with CCR5 inhibitor. This compound reduces CCR5^+^ T-reg cells in the tumour microenvironment increasing the immune response and cancer regression [71, 72].

## Conclusion

The last decades have seen the accumulation of a huge amount of cancer genomics data, that have supported the development of predictive models of cancer [71]. Despite this data have undoubtedly pointed out a role for BAP1 in tumorigenesis of a specific spectrum of cancer, the determination of the molecular underlying mechanisms has not yet led to development of therapeutic strategies. Important questions remain elusive; the selectivity of the cancer spectrum has not yet an explanation. Remarkably the relevance of the epigenetic role of BAP1 versus the other described functions has still no answer. Finally, we argue that in cancer development the maintenance of genomic integrity is a pivotal aspect of the pathogenesis and whether and BAP1 dependent regulation of the cellular epigenome impact the genomic integrity remain an eluded critical question.
